# Spatial malaria epidemiology in Bangladeshi highlands

**DOI:** 10.1186/1475-2875-8-185

**Published:** 2009-08-05

**Authors:** Ubydul Haque, Mamun Huda, Awlad Hossain, Syed Masud Ahmed, Mohammad Moniruzzaman, Rashidul Haque

**Affiliations:** 1 International Center for Diarrhoeal Disease Research Bangladesh, 68 Shaheed Tajuddin Ahmed Sharani, Mohakhali, Dhaka 1212, (GPO Box 128, Dhaka 1000), Bangladesh; 2 BRAC, BRAC Centre, 75 Mohakhali, Dhaka 1212, Bangladesh; 3 Local Government and Engineering Department, GIS Unit, Agargaon, Dhaka, Bangladesh

## Abstract

**Background:**

Malaria is a major public health burden in the south-eastern part of Bangladesh, particularly in the Chittagong Hill Tracts region. In 2007, BRAC and ICDDR,B carried out a malaria prevalence survey in the endemic regions including the Khagrachari District.

**Methods:**

This study was done to detect clusters of malaria and identify the geographic risk factors. Thirty mauzas (the lowest administrative unit/bigger than village in Bangladesh that has polygon boundary) from the area were selected for the survey using probability proportional to size (PPS) sampling. Twenty-five households within each mauza were then randomly selected for screening, with a GPS point being recorded at each household. Rapid diagnostic tests were used to diagnose malaria.

**Results:**

The average malaria prevalence in the District was 15.47% (n = 750). SaTScan detected five geographic clusters of malaria, one of which was highly significant (p = 0.001). Malaria cases were significantly associated with proximity to water bodies and forests.

**Conclusion:**

The data presented in this paper are the first step to understanding malaria in southeastern Bangladesh from a micro-geographic perspective. The study results suggest that there are 'malaria hot-spots' in the study area. The government of Bangladesh and non-governmental organizations involved in malaria control should consider these results when planning malaria control measures. In particular, malaria maps should be updated on a regular basis as new data become available.

## Background

Malaria is a major health burden in the south-eastern part of Bangladesh, especially in the Chittagong Hill Tracts [1]. In 13 endemic districts, total malaria prevalence was 3.97% with the rapid diagnosis test (BRAC and ICDDR,B unpublished report). To control malaria in hilly forest areas is a big challenge in many parts of Asia and South America [2].

Disease mapping techniques help to control malaria, especially in high malaria transmission areas. In order to control malaria, application of GIS and GPS was reported in India, Thailand and Madagascar. GIS was used to detect high risk areas and for malaria surveillance in India. In Thailand, GIS has proven to be a significant tool for forecasting malaria epidemics [3]. These statistical techniques depend on case event and count data, where geographic locations (x, y coordinates) are represented as points. Recently, Geographic information system (GIS) and remote sensing (RS) technology have enabled scientists and epidemiologists to study vector borne disease, mapping, to explore environmental relations, and to understand spatial and temporal distribution [4].

Mosquito vectors breed near rivers, pools, agricultural lands and forests; they depend on the existence of water and river flow [5]. Malaria risk maps were developed in Sri Lanka based on the household distance from streams and rivers that are known as potential vector breeding sites. People living within 750 meters of streams were identified as high-risk groups [6]. In southern Ontario, Canada, SaTScan was used to investigate spatial clusters of giardiasis. SaTScan successfully identified approximate locations and spatial clusters [7]. Recently GIS, GPS and spatial statistics have also been used in malaria research and control in sub-Saharan African countries. Malaria maps have long been considered an important tool to control malaria. Generally this type of map is used to predict risk [8].

In order to detect aggregation of disease cases, cluster detection is very important. Until now, there is no published report on the micro-geographic distribution of malaria incidence or prevalence in Bangladesh. This study uses GIS, GPS, and SaTScan tools to detect geographic clusters of malaria, to assess association between malaria cases and geographic risk factors.

## Methods

### Data collection and data preparation

The population figures from 2001 population census of Bangladesh were used for sampling [9]. Multi-stage cluster sampling technique was used. Sample size was calculated using web-based software C-Survey 2.0 based on the conservative estimates of malaria prevalence and design effect. In Khagrachari, all mauzas were listed and 30 mauzas (the lowest administrative unit of Bangladesh that have polygon boundary) were selected using a probability proportional to size (PPS) sampling procedure. These mauzas are the study clusters. City corporations and towns were not included in this survey. Thirty mauzas were selected for this survey. Twenty-five households were selected using systematic randomization from each mauza. In each cluster, the study team drew a map. Households were then chosen through a systematic random sampling as mentioned above. Simple random sampling was then used to select one individual from each household. This individual was screened using rapid diagnostic tests for malaria after obtaining written informed consent from the individual or their legal guardian.

### Rapid diagnostic tests

Malaria was diagnosed by using rapid diagnostic tests (RDT, FalciVax) to detect *Plasmodium falciparum *and *Plasmodium vivax*-specific antigens. The trade name of this RDT is "FalciVax" and is produced by Zephyr Biomedicals, India . Each FalciVax is rapid self-performing, qualitative, two site sandwich immunoassay utilizing whole blood for the detection of *P. falciparum*-specific histidine rich protein-2 (Pf, HRP-2) and *P. vivax*-specific pLDH. The test can be used for specific detection and differentiation of *P. falciparum *and *P. vivax *malaria. The standardization of this test has already been done by the Zephyr Biomedicals. Sensitivity of the RDT is similar to that commonly achieved by good field microscopy. Sensitivity and specificity of the RDT used for the detection of *P. falciparum *and *P. vivax *is more than 95% and now been recommended for use in the malaria control programme by the World Health Organization [10-12]. A recent study in India also confirmed the reliability of FalciVax to diagnosis malaria [13].

### GPS data collections

**Figure 1:**
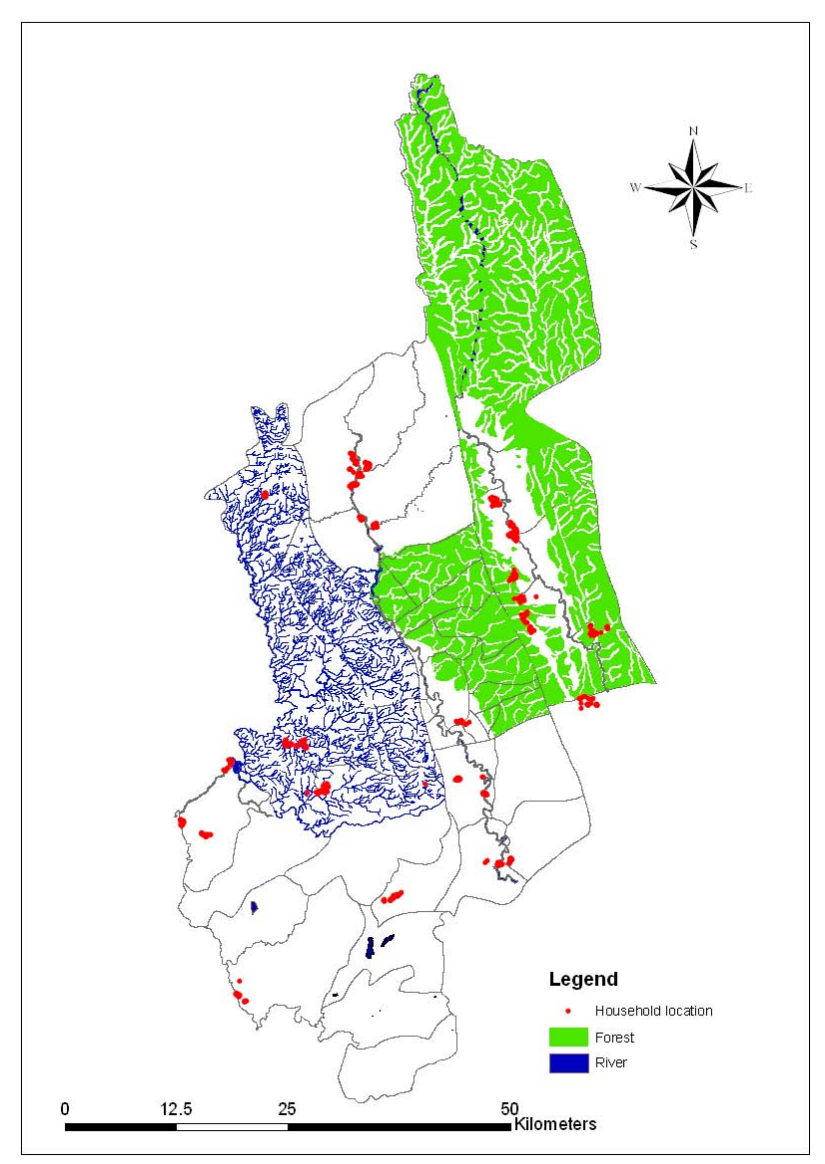
**Land use map of Khagrachari**.



The coordinates (longitude and latitude) of all selected households (n = 750) were recorded on-site using eTrex Venture single handheld GPS receivers. GPS points were uploaded to a Fox Pro database system and cleaned for duplicates. Household positions were printed in hard copy and accuracy was checked at the field level. Water and forest data were obtained from the Local Government and Engineering Department (LGED) of the Government of Bangladesh (figure 1). Distances between points of interest were calculated using the following equation:

### Study area and population

Khagrachari is a hilly area covered with forests, lakes and streams that have provided an excellent breeding ground for malaria vector. It is surrounded by Tripura (Indian state) to the north, Rangamati and Chittagong to the south, Rangamati to the east and Chittagong and Tripura to the west. Khagrachari has a total area of 2,699.55 square kilometer and a total population of 524,961. The temperature ranges from 13°C to 34.6°C and annual rainfall is 3,031 mm.

### Data analysis

Epidemiologic information and laboratory results were linked to each household's GPS data. Maps were produced with Arc GIS 9.1 software. SaTScan (v. 07) was used to detect spatial clusters (settings: spatial analysis; Bernoulli probability model; Cartesian coordinates; no geographical overlap). Clusters were determined by calculating the maximum likelihood ratio. Standardized incidence ratios (SIR) were estimated by dividing the number of observed cases by the number of expected cases in each cluster. Simulated p-values were obtained using Monte Carlo methods with 9,999 replications. Statistical analyses were done with SPSS 11.5 software. Multiple logistic regression models were used to control for confounding.

## Results

**Figure 2:**
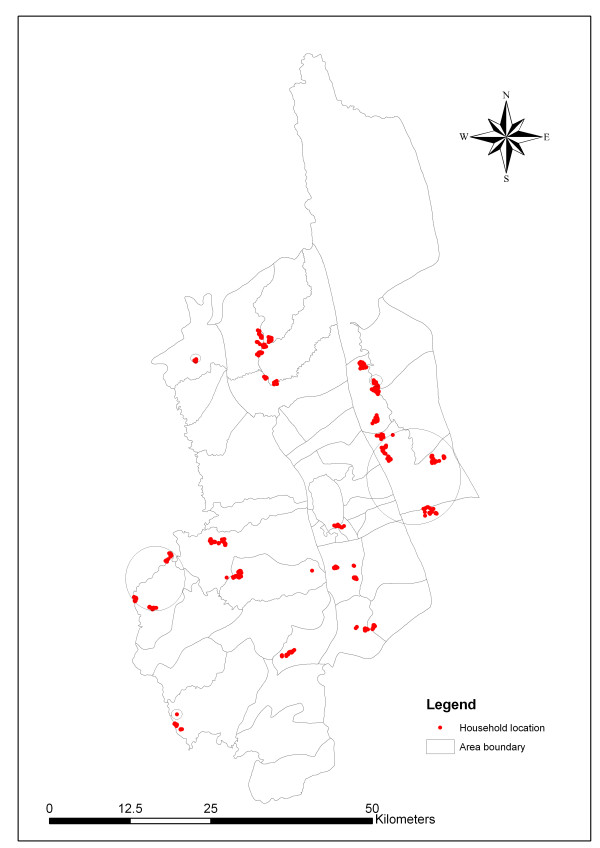
**Distribution of malaria clusters in Khagrachari**.

The average malaria prevalence in Khagrachari district was 15.47% (n = 750). [District is divided into several thanas. Thanas are again divided into unions. Again unions are divided into mauzas the lowest administrative unit in Bangladesh]. Table 1 lists the individual prevalence of malaria for the thanas in Khagrachari. The highest prevalence was found in Dighinala (22%) and lowest prevalence was found in Panchari (5.72%). SaTScan was used to detect malaria clusters in Khagrachari (n = 750). Table 2 presents there were five clusters in total. Among them, one was most likely clustered and four were secondary clusters (figure 2). The most likely malaria case cluster area is in Dighinala thana (RR = 3.381; p = 0.0002). In most of the secondary clusters, the relative risk ratio was high, but none were statistically significant.

**Table 1:** Malaria prevalence in Khagrachari district

**Thana**	**Prevalence (%)**
Dighinala	22

Panchari	5.72

Manikchari	8

Khagrachari sador	10

Mahalchari	16

Ramgarh	21.34

Matiranga	21.6

**Table 2:** Spatial malaria clusters in Khagrachari district detected by SaTScan v7.0.3

Cluster	Population	No. of Cases	Expected Cases	Relative Risk	Log Likelihood Ratio	P-Value
Most likely cluster						

	85	35	13.15	3.381	19.066555	0.0002

Secondary clusters						

	73	24	11.29	2.419	7.742381	0.0824

	3	3	0.46	6.611	5.632573	0.8629

	3	3	0.46	6.611	5.632573	0.8629

	5	4	0.77	5.321	5.185802	0.9100

Table 3 presents the distribution of environmental and socioeconomic characteristics by malaria positive, negative and total household. Table 4 lists the adjusted odds ratios for malaria based on the distance from each participating household to various locations, including forest, and water sources. A binary variable with 3 km as the cut-off point was created because most mosquitoes cannot fly more than 3 km from their breeding places [14]. Results reveal that people living in houses less than 3 km from the forest were at much higher risk for malaria than people living further away. The strong effect of distance from house to forest remained significant after controlling for a number of potentially confounding variables. People living in houses more than 3 km from the water sources were at much higher risk of malaria than people living within 3 km. After controlling for confounding by other variables, the effect remained significant for the distance from house to water sources. Children had a significantly higher risk for malaria than adults. Independent risk factors for malaria infection found significant in multivariate analysis for the entire surveyed area were proximity to forest, precarious house (house made with temporary construction materials like straw, polythene, bamboo etc), and bed net with <3 in house. Only proximity to water came out as an independent and significant protective factor for malaria infection.

**Table 3:** Distribution of environmental and socioeconomic characteristics by malaria positive, negative and total household (HH)

	**Malaria Positive household** **N = 116**		**Malaria Negative household** **N = 634**		**Total household** **N = 750**	
		
	**%**	**95% CI**	**%**	**95% CI**	**%**	**95% CI**
		
**Forest within 3 km**	37.9	29.1-46.8	29.5	26.0-33.1	30.8	27.5-34.1
	
**Water within 3 km**	55.2	46.1-64.2	72.4	68.9-75.9	69.7	66.4-73.0
	
**Precarious house**	46.6	37.5-55.6	28.5	25.0-32.0	31.3	28.0-34.7
	
**HH with less than 3 bed nets**	62.9	54.1-71.7	46.1	42.2-49.9	48.7	45.1-52.3
	
**Having cattle or small animal^$^**	95.7	91.9-99.3	92.4	90.4-94.5	92.9	91.1-94.8
	
**HH with more than 5 family members**	41.4	32.4-50.3	33.6	29.9-37.3	34.8	31.4-38.2
	
**Illiterate household head**	50.0	41.0-59.1	45.6	41.7-49.5	46.3	42.7-49.8
	
**Deficient HH**	62.1	53.2-71.0	50.8	46.9-54.7	52.5	48.9-56.1

^$^Chickens, ducks, goats

**Table 4:** Risk factors for malaria in Khagrachari, Bangladesh

	**Crude OR**	**(95% CI)**	**Adjusted OR**	**(95% CI)**	**P-value**
**Distance to forest**					
- **< = 3 km**	1.50	(1.00-2.21)	2.23	(1.37-3.60)	0.001
- **>3 km**	1.00		1.00		

**Distance to water**					
- **≤3 km**	0.47	(0.31-0.70)	0.50	(0.31-0.80)	0.004
- **> 3 km**	1.00		1.00		

**Housing status**					
- **Precarious**	2.18	(1.50-3.30)	1.66	(1.04-2.64)	0.034
- **Non-precarious**	1.00		1.00		

**Bed net in house**					
- **<3 bed net**	2.00	(1.30-3.00)	1.70	(1.10-2.72)	0.028
- **≥3 bed net**	1.00		1.00		

**Age**					
- **≤17 years**	4.27	(2.83-6.45)	4.14	(2.71-6.32)	<0.0001
- **> 17 years**	1.00		1.00		

## Discussion

Malaria epidemiology was never studied in the study area before. The prevalence study showed a high prevalence rate. This is also the first data set and can be useful for target interventions for control programme. Malaria is primarily clustered in Dighinala, which shares a common border with India and Myanmar, and has hilly reserve forests and water bodies. This results originated from the cross-sectional study. Malaria clusters may differ because of seasonal variation. The result is very important because there are several malaria clusters even in a high endemic area. Government and BRAC who is implementing the national malaria control programme should update passive surveillance data and detect malaria cluster in regular basis for target intervention. That will provide an opportunity of optimum use of limited resources for the national malaria control programme.

Individual and household level risk factors were investigated. Logistic regression was used to examine the effects of distance to forest and water bodies, housing status, bed net use and age. Consistent with other studies proximity to forest was found to be a risk factor for malaria in the study region [15]. In contrast to common findings the proximity of household to water bodies was found to be protective against malaria. These unusual findings were discussed with international malaria experts and they were not surprised by the findings noting that such findings have occurred in other locations. One explanation offered was that people living closer to breeding sites are more aware of malaria and thus take more precautionary measures than others.

Housing status has been shown to be an important risk factor of malaria. In Burkina Faso, *P. falciparum *prevalence was two times higher for those living in mud-roofed houses compared to those living in iron-sheet roofed houses compared [16]. A similar study in Sri Lanka showed a strong significant relationship with poorer housing construction and the presence of indoor-resting mosquitoes [17]. In this study, precarious housing was associated with malaria risk. Additionally, significant risk was associated with those in households possessing less than three bed nets, which is widely supported in the literature [2,18]. Unsurprisingly children in the Khagrachari district are at significantly greater risk of contracting malaria.

This study also suggests the target interventions in the high risk areas that can help significantly to control malaria. Through the map it is possible to determine which areas require the greatest control effort. The maps presented in this paper are the first step to understanding malaria in hilly Khagrachari from a micro-geographic perspective.

## Conclusion

Understanding the spatial distribution of malaria, identifying geographic risk factors and the population at risk are important steps toward effective control of malaria. The data presented in this paper are the first step to understanding malaria in south-eastern Bangladesh from a micro-geographic perspective. The study result suggests that there are 'malaria hot-spots' in the study area. The government of Bangladesh and non-governmental organizations involved in malaria control should consider these results when planning malaria control measures. More efforts should be focused on people living in remote areas. BRAC the largest NGO in Bangladesh is currently implementing malaria control programme in Bangladesh. They have deployed health workers in grass root level. They have supplied ITN (Insecticide treated net) in every household. They should consider this result to control malaria and investigate the reasons of cluster.

They should be provided with more bed nets and anti-malarial drugs. One way to accomplish this would be to redistribute health workers to remote regions. The government should also consider allocating additional resources so that more operational research can be carried out in the micro-geographic area. In particular, malaria maps should be updated on a regular basis as new data become available.

## Competing interests

The authors declare that they have no competing interests.

## Authors' contributions

UH designed, analysed and prepared the manuscript, AH collected data, MH analysed the data, RH and SMA were responsible for study design and conduct the study.
